# Unpacking complexity in addressing the contribution of trauma to women’s ill health: a qualitative study of perspectives from general practice

**DOI:** 10.3399/BJGP.2024.0024

**Published:** 2024-08-06

**Authors:** Jennifer MacLellan, Sharon Dixon, Francine Toye, Abigail McNiven

**Affiliations:** Nuffield Department of Primary Care Health Sciences, University of Oxford, Oxford.; Nuffield Department of Primary Care Health Sciences, University of Oxford, Oxford.; Physiotherapy Research Unit, Oxford University Hospitals NHS Foundation Trust, Oxford.; Nuffield Department of Primary Care Health Sciences, University of Oxford, Oxford.

**Keywords:** biopsychosocial models, communication, complexity, general practice, trauma-informed care

## Abstract

**Background:**

There is an intricate relationship between the mind and the body in experiences of health and wellbeing. This can result in complexity of both symptom presentation and experience. Although the contribution of life trauma to illness experience is well described, this is not always fully recognised or addressed in healthcare encounters. Negotiating effective and acceptable trauma-informed conversations can be difficult for clinicians and patients.

**Aim:**

To explore the experience of primary care practitioners caring for women through a trauma-informed care lens.

**Design and setting:**

Qualitative study in the general practice setting of England, with reflections from representatives of a group with lived experience of trauma.

**Method:**

This was a secondary thematic analysis of 46 qualitative interviews conducted online/by telephone to explore primary care practitioners’ experiences of supporting women’s health needs in general practice, alongside consultation with representatives of a lived-experience group to contextualise the findings.

**Results:**

Four themes were constructed: ‘you prioritise physical symptoms because you don’t want to miss something’; you do not want to alienate people by saying the wrong thing; the system needs to support trauma-informed care; and delivering trauma-informed care takes work that can have an impact on practitioners.

**Conclusion:**

Primary care practitioners are aware of the difficulties in discussing the interface between trauma and illness with patients, and request support and guidance in how to negotiate this supportively. Lack of support for practitioners moves the focus of trauma-informed care from a whole-systems approach towards individual clinician–patient interactions.

## Introduction

As evident in the Women’s Health Strategy for England^1^ and its underlying public consultation,^2^ women’s health is complex and embedded in historical dismissal and stigma. There is an intricate relationship between the mind and the body in experiences of health and wellbeing. One facet of this complexity includes the possible contribution of trauma to the woman’s illness experience. The physical response to, and pathways of bodily damage as a result of, the hormonal environment of chronic stress has revealed links between unresolved emotional distress and autoimmune conditions.^3^ Trauma has an impact on people in different ways. Although some people make positive adjustments, others experience mental ill health and/or develop physical symptoms from emotional distress.^4^ This can result in complexity both in symptom presentation and health experience.

Trauma can result from an event, series of events, or set of circumstances that is experienced by an individual as harmful or life threatening and can include past experiences of care (including in maternity), adverse childhood events (ACEs), and other life experiences as an adult. ACEs are stressful or traumatic events that occur specifically during childhood or adolescence^5^ and can include: abuse (physical, emotional, and sexual); neglect; living in a household with domestic violence, experience of illness, or bereavement.^6^ In a systematic review and meta-analysis of 96 studies of adult health behaviours, the risk of poorer health outcomes (including cardiovascular disease, respiratory disorders, gastrointestinal disorders, and mental ill health) increased with the number of ACEs.^4^ Experiences of trauma at any stage in life can cause lasting adverse effects on health.^3^ In the UK, women are disproportionally affected by violence (twice as likely as men to experience domestic violence),^7^^,^^8^ trauma,^9^^,^^10^ and ill health,^11^^,^^12^ highlighting the potential complexity of women’s health presentation.

Although the contribution of life trauma to illness experience is well described, primary care professionals do not always fully address it. Potential reasons include clinician concerns about missing a serious illness in a complex presentation or about alienating or upsetting the patient.^11^ Addressing trauma often necessitates introducing conversations about the link between mind and body, which can be difficult to navigate. Significant challenges and uncertainties reside in how best to manage the link between mind and body in communication with patients and in healthcare pathways. Qualitative research indicates that primary care professionals can find it challenging to navigate this mind–body presentation. Suggestions from primary care professionals that physical symptoms are amplified by (or a manifestation of) distress can be experienced as dismissal and invalidation by patients.^13^^–^^15^ Attempts to bridge these health needs are therefore not always experienced as supportive. This illustrates the potential challenges of negotiating trauma-informed conversations in ways that are experienced as acceptable and supportive by patients.

**Table 1:** How this fits in

Significant challenges and uncertainties reside in how best to manage the link between mind and body in communication with patients and in healthcare pathways. Lack of supportive resources to deliver holistic, trauma-informed care risks practitioners (inadvertently) avoiding discussion of the contribution of distress in the illness presentation. A trauma-informed systems-level approach would support integration of psychological support within multiple care pathways and support wellbeing of practitioners providing care.

Trauma-informed care is a framework founded on five core practices: safety, trustworthiness, choice, collaboration, and empowerment. These can be used to address the impact of trauma on patients and healthcare professionals and prevent re-traumatisation in healthcare services.^16^ However, definitions, guidance, practitioner training, delivery, and support for trauma-informed approaches vary between healthcare settings according to local-level funding priorities with implementation described as disjointed.^16^ Little is known about how healthcare professionals experience trying to effectively deliver trauma-informed care. The aim of this study was to explore the experiences of primary care practitioners caring for women through a trauma-informed care lens.

## Method

This study was a secondary analysis of qualitative interview data gathered to explore primary care practitioners’ experiences of supporting women’s health needs in primary care. Between March and September 2022, we interviewed a sample of 46 primary care practitioners across England (GPs *n* = 31, nurses *n* = 9, other professionals *n* = 6, with an average of 12 years’ experience [1 to 30 years], 41/46 female), ensuring representation from practices working in areas of deprivation where health inequalities and multimorbidity are significant challenges. Detailed methods and participant characteristics of the parent study are reported elsewhere.^17^

The original topic guide was developed by three authors in response to a perceived gap in knowledge about women’s health care in primary care and commissioned by the National Institute of Health Research (NIHR) Policy Research Programme. Data were collected through single-episode, one-to-one interviews with fully informed consent. They were conducted virtually online or by telephone by two experienced qualitative researchers and audio-recorded. These were transcribed verbatim, checked against the original recording, and thematically analysed.

The team then undertook a focused enquiry using secondary thematic analysis of the dataset to explore primary care professionals’ navigation of women’s experiences of distress as a contribution to their symptoms.^18^ We recoded the transcripts line-by-line where distress, emotional, or psychological impact or contribution to health experience was mentioned. We discussed the constructed data categories within the research team to create interpretive themes. We reflected on these themes with representatives of three charities supporting women with significant experience of historical and contemporary trauma to add a lived-experience perspective to the data.

## Results

Four themes were constructed from the data:

- ‘you prioritise physical symptoms because you don’t want to miss something’;
- you do not want to alienate people by saying the wrong thing;
- the system needs to support trauma-informed care; and
- delivering trauma-informed care takes work that can have an impact on practitioners.

### Theme 1: ‘you prioritise physical symptoms because you don’t want to miss something’ (PC30, female [F], GP for 5 years)

Practitioners described women’s health consultations as often complex and difficult to manage in a single, constrained time slot. A significant concern was the fear of missing a physical condition requiring specific or prompt treatment as many women’s health complaints could present with similar but vague symptomatology and could suggest multiple possible diagnoses. Some participants reflected that a challenge of navigating diagnostic processes, by first excluding potential causes that need specific interventions such as cancer, meant the contribution of distress to physical symptoms was pushed down the list of considerations:

*‘It’s definitely sort of a symptom sieve to start with, and to adequately hear your patient and really hear them and really listen to what they’re saying* […] *There are many things that are difficult to do in ten minutes, but I … women’s health is particularly difficult.’*

(PC17, F, advanced nurse practitioner [ANP] for more than 15 years)

*‘They’re often quite vague symptoms: bloating, things like that, so you either have a very low index of suspicion and you’re seeing ca-125s* [blood test that may indicate ovarian cancer] *and you’re scanning everybody, or things get missed, and* [sighs] *yeah, it can be very challenging and obviously if you miss something like that it’s devastating for everybody involved, but it’s very difficult.’*

(PC12, F, GP for more than 15 years)

Participants described how investigation pathways move through a hierarchy of potential causes and may involve a stepped process that did not always yield a confirmatory or unifying diagnosis. This meant that the participants had to manage patients’ expectations of diagnosis throughout this process.

### Theme 2: you do not want to alienate people by saying the wrong thing

Some felt that a cultural shift was needed for the wider healthcare system to acknowledge the mind–body interplay as a legitimate expression of distress, to support practitioners to discuss this with their patients along their care pathway, and to provide timely access to psychological support services:

*‘Perhaps some of training for staff would be about how you talk about the connection between your brain and your body* […] *without sounding dismissive and actually, training individuals to become more sensitive to these types of, conversations.’*

(PC46, male [M], GP for 15 years)

However, some felt that patients were not always receptive to recognising the contribution of emotions or past experiences to physical symptoms, the idea of an integral link between mind and body, or the offer of psychological support to cope with the distress of physical symptoms. Some participants were worried about alienating women who might interpret this suggestion as devaluing or de-legitimising their symptom experience, and were therefore sometimes unsure when or how to navigate this:

*‘I don’t think many patients like it when we end up going down that route when it comes to pain, any pain, not just pelvic pain in itself, because they want a diagnosis of some form or another, whatever it’s called, rather than being given some antidepressants or some counselling.’*

(PC18, F, GP for 10 years)

Participants described the essential first step to be validation of the woman’s experience, emphasising understanding and genuine belief in the symptoms as ‘real’ (although perhaps currently unexplained) before exploring the impact of trauma or life stress in its aetiology:

*‘It’s just spending the time with them and actually acknowledging, yes the pain is real, but are we not just saying you know, “you’ve got pain and we can’t find any cause for it”, “the pain is actually real”, and what we can do is maybe go down the route of psychological sort of therapy for that, that might be the best route of managing it.’*

(PC18, F, GP for 10 years)

*‘The first lady I was talking about absolutely wasn’t having any of it* […] *I got her some interesting resources* […] *and I just mis-pitched it* […] *the fact that this is her body feeling overwhelmed and feeling overwhelmed with the difficulties in her life and how to explain that in a way that seems scientific … it’s quite difficult, isn’t it?’*

(PC14, F, GP for 1 year)

Healthcare professionals were aware and worried that exploring the contribution of trauma or distress in the physical symptom experience and that physical and emotional symptoms can coexist was not always well received. Restricted time in consultations highlighted the need for resources that could support this mind–body understanding in a positive and affirming way for the patient:

*‘Often there is something organic, or something organic that has started it off, but then it often becomes this kind of complex combination of physical and then also psychological symptoms together, and I think kind of having resources to explain how psychological symptoms can impact pelvic pain* […] *I think kind of having good resources to try and back up what I’m saying would be quite helpful.’*

(PC21, F, GP for more than 20 years)

Participants described how the net effect of these considerations could result in practitioners (inadvertently) avoiding discussion of the contribution of distress in the illness presentation:

*‘*[…] *I think you can shut it down easily and not get emotionally involved, but you do not actually solve any of the issues unless they are straight up, simple, physical problems that you can just treat, but for the most part it doesn’t work very well.’*

(PC30, F, GP for 5 years)

Participants recognised the importance of a trauma-informed approach in the complex and holistic care needs of women’s health. This extended to considerations about trauma-informed approaches to physical examination and how this could be enabled. Some highlighted the unique position of the primary care practitioner, in a potentially protracted diagnostic or support pathway, to communicate the contribution of distress in a supportive and helpful way to their patients.

### Theme 3: the system needs to support trauma-informed care

Participants described four systemic challenges to the provision of trauma-informed care:

- inadequate time allocated for appointments;
- waiting times for specialist practitioner review in secondary care;
- limited access to services; and
- providing care for women returning from secondary care without a unifying diagnosis.

The challenges of time were frequently reported by participants:

*‘I already know that I can’t do everything for you* [the patient] *in ten minutes, which isn’t always like a nice feeling for me, because we want to be able to help and you know do that within the time … who knows when they’ll be able to get an appointment again or you don’t want it to be frustrating for them, but equally you don’t want to rush yourself.’*

(PC35, F, GP for less than 6 months)

*‘They come back two months later and say, “I’ve still … I’m still … still haven’t seen the hospital”, and that there’s a certain amount of workload in primary care just because of … just because secondary care can’t take that on.’*

(PC23, M, GP for more than 20 years)

In some areas they reported limited access to services such as counselling or psychological support services and community gynaecology because of local funding models and the challenges of providing care for women returning from secondary care without a unifying diagnosis. This often led to practitioners ‘holding the distress’ of the woman (see theme 4). Despite the challenges identified, participants described how they worked within the system constraints to offer the best service for their patients, for example, planning activities across multiple appointments:

*‘In fifteen minutes it’s quite challenging, or if I’m trying to examine somebody* […] *that’s difficult, that’s when I sometimes ask them* […] *to come back for the examination so that I can do all the other things that are needed.’*

(PC25, F, GP for 25 years)

Participants spoke of the structural supports that were in place that worked well in their efforts to deliver trauma-informed care, such as support networks, the ‘advice and guidance’ contact service to access secondary care (a system where GPs can access specialist advice before or instead of referral), and working with social prescribers (link workers who help patients to access non-medical support services in their community):

*‘I mean advice and guidance* [are] *probably helpful I think, you write and you say, “What do I do?” and they tell you, and you then say to the patient, “this is what the specialist has said”, and that’s great, and that’s a really good idea.’*

(PC23, M, GP for more than 20 years)

‘[Access to a social prescriber] *is definitely making a difference; I don’t know what we did before to be quite honest. I don’t know what we would do because it’s just improved the quality of life for our patients, and it’s just helped us cope because you know we often see mental health problems, social problems, and with such a limited time constraint, limited resources, now that investment has been put in, it is definitely making a difference.’*

(PC16, F, ANP for more than 18 years)

### Theme 4: delivering trauma-informed care takes work and can have an impact on practitioners

Taking a trauma-informed approach relied heavily on the practitioner–patient relationship and some felt that the impact on practitioners was not always accounted for. The work involved in taking a trauma-informed approach to care had an impact on clinician workload. When they were able to navigate this challenge participants reported job satisfaction that was a positive impact. Conversely, when participants were unable to deliver the care they aspired to and believed they should, this had a negative impact. Protracted routes to diagnosis (or not getting a diagnosis), exacerbated by long waits to access specialist review in secondary care, left participants ‘holding the distress’ of women managing symptoms while they waited for a management plan:

*‘I mean typically what happens is when a referral is done, the patient is waiting three, four, five months to be seen sometimes, but the patient’s still got those symptoms, so what do they do?’*

(PC18, F, GP for 10 years)

*‘So pain is complex. I think every pain service in the country is poorly funded and poorly accessible* […] *The challenge we have is these patients are constantly accessing us and, you know, I don’t want to label anything but they do end up becoming frequent attenders, which you know … and all we are is becoming a holding person in all of this.’*

(PC46, M, GP for 15 years)

This increased the pressure on primary care practitioners who were operating without adequate system support. Although participants knew that managing uncertainty was integral to the role of the primary care practitioner, holding distress added to the challenge of appropriately broaching or exploring the mind–body link. Participants described feeling overwhelmed and personally affected by managing the expectations of patients held in limbo and holding their distress:

*‘Women who have complex, like intractable symptoms that have been investigated and no one’s really come up with anything* […] *it’s more psycho-social input that’s needed, and they’ve seen a gynaecologist and they’re still struggling and there’s not really a solution, and so they’re … they’re the ones who you think, “oh my gosh, I … I’m … I’m not sure what I can offer … offer you”.’*

(PC34, F, GP for 15 years)

*‘I mean women’s health is a prime one, it causes so much anxiety, stress, impact on the family, and I think with the complexities around the referral pathways and who’s doing what, which has been one of my biggest stresses, people can fall through the gaps very easily.’*

(PC26, F, GP for 5 years)

Participants sought support from colleagues within their daily work routines to reflect on clinical questions or patients with complex cases. However, some felt that there were limited support services for practitioners’ mental wellbeing in a more formalised and structured way:

*‘We have our annual appraisal but that is very much to make sure that we’re not total lunatics* […] *but other than that* […] *they do support us, but they … you know it’s once a year, there’s no capacity to debrief on individual challenging cases or anything like that, it’s very much to check-in that we are sort of on the rails.’*

(PC30, F, GP for 5 years)

Participants described how not being able to deliver high-quality, holistic care because of structural constraints was unsatisfying and challenging:

*‘I was so unhappy in my previous job really, I’d say we still had support, but the patients were a lot more demanding and it just comes with that, you know a lot more child protection issues safeguarding and it … you know, it’s just a really challenging job and that, and not necessary work satisfying either.’*

(PC04, F, GP for 3 years)

Lack of personal and systems support for practitioners moves the focus of trauma-informed care from a whole-systems approach to the clinician–patient interaction.

## Discussion

### Summary

Our findings indicate that clinicians are aware of the contribution of trauma and distress to the presentation of physical symptomatology within women’s health consultations but that conversations about this could be difficult. Some participants felt confident and willing to discuss the role of distress in symptom presentation; others felt that these conversations were difficult and sometimes avoided the topic. Constraints such as limited time in consultations and the training and resources to facilitate discussions about the minded-body (the interconnection of physical and emotional health) and the role of trauma and distress could mean that clinicians did not always talk to patients about the impact of distress. This was exacerbated by system constraints such as limited support services for referral. Practitioners described building support mechanisms for themselves at work through debrief and clinical conversations with colleagues but told us that there were no formal supervision or support services routinely available for practitioners. The heavy work and emotional labour within an unsupportive system was described as contributing to practitioner frustration and burnout. Although patient relationships were framed within a trauma-informed lens, the organisational configuration was not always supportive to a trauma-informed approach.

### Strengths and limitations

The use of secondary analysis has allowed us to conduct a focused analysis on a rich dataset of primary care professionals’ interviews. As this was done within the project timeline by the original research team, potential ethical concerns about the impact of the sociopolitical context that often accompanies secondary analysis were mitigated.^18^ We were able to minimise participant burden and engage with a targeted group of women for whom trauma-informed care and its delivery has an immediate impact.

The principal limitation of our study is the restrictions offered by the original interview scope and guiding questions of the parent study that focused on women’s health. We are unable to report on experience in other areas of health care or by gender of care provider as this is unexplored. Gender was recorded; there were four male and 42 female responders. We purposively selected practitioners with an interest in women’s health rather than sampling an equally gender-split sample to derive patterns of experience that could be attributed to gender issues.

### Comparison with existing literature

The link between trauma and ill health is well discussed in the literature, as are the principles of trauma-informed care. However, there appears to be little evidence of the clinician’s experience in discussing the interface between trauma and complexity with patients. The complexity of women’s health experiences challenges a dualistic approach to care and could respond better to the continuity model of primary care.^19^ Practitioners in our data actively enacted the principles of trauma-informed care (such as safety, trustworthiness, and collaboration) in their personal practice with women.^16^ However, the structural configuration of primary care services could complicate these care aspirations including when resources were limited or services were not flexible enough to support practitioner autonomy, which could hinder opportunities for timely care or follow-up. This could erode the practitioner’s efforts to deliver trauma-informed care, with potential consequences for both patients and clinicians. Such structural constraints in a climate of overwork are powerful sources of moral distress and burnout in studies of nurses, midwives, and doctors.^20^^–^^23^ The risk of exposing practitioners to such moral distress can lead to the experience of vicarious trauma and reduced job satisfaction as they navigate the challenge of exploring the minded-body link with patients on their illness journey.^24^^,^^25^ Primary care practitioners held women’s distress while they waited for specific therapies or supports, and yet the practitioners did not have adequate formal support systems to take care of their own wellbeing. This finding resonates with Pereira Gray *et al*,^25^ who suggest that the UK shortage of GPs, erosion of continuity of care, sustained increase of remote consultation methods, and lack of structural support in the system may exacerbate challenges faced by practitioners to provide high-quality care.^26^^–^^28^

### Implications for research and practice

Our findings suggest that moving towards a trauma-informed systems-level approach would support integration of psychological support within multiple care pathways. A coordinated systems approach should support an integrated and holistic approach rather than encouraging a dichotomising split between physical or psychological services. Our findings suggest that this model would also support the wellbeing of practitioners delivering care and may have an impact on staff retention, making this a critical consideration at all system and service levels from individuals to practices to funders and commissioners.^28^^,^^29^ However, less is known about how to enact or enable trauma-informed care at a systems level.^16^ More research is needed about how to implement and support equitable, proportionate trauma-informed care in practice. This includes learning how to actively nurture equitable care within services, practices, and within primary care networks. At a funding and commissioning level, autonomy and equitable work need to be valued and enabled, and this requires policy attention; simplistic metrics of care such as numbers seen or a narrow focus on numerically quantifiable access will not capture either the impact on patients or practitioners.^28^ Nor will this capture the contacts and appointments that did not happen. Furthermore, critical to effective equitable care is that practitioners need meaningful access to services that they can refer into and that will respond promptly and supportively to the needs identified. Work in areas of care such as female genital mutilation and domestic violence and abuse demonstrate that having acceptable accessible services to refer into enabled inquiry and compassionate care.^30^^,^^31^ It is an ethical prerogative that trauma-informed enquiry is supported by trauma-informed services and support. Finally, support for staff is essential and the responsibility for this should not be devolved to individuals but commissioned and provided for. This contrasts with current policy, such as the wellbeing Quality and Outcomes Framework indicators that arguably devolve the responsibility for wellbeing to those in need of wellbeing support, without offering any tangible resources.

Healthcare professionals are aware of the difficulties in discussing the interface between trauma and complexity with patients^32^ and our work shows they are requesting support and guidance in how to negotiate this supportively. The British Medical Association moral injury report^22^ recommends systems changes that map onto the principles of trauma-informed care, including increased staffing, streamlining of bureaucracy, open and sharing work cultures, and provision of support for employees. However, although these recommendations acknowledge the problem and offer solutions, there is no requirement for organisations to address these structural concerns. Lack of these system supports for practitioners moves the focus of trauma-informed care from a whole-systems approach to the clinician–patient interaction.^16^

To seek lived-experience perspectives on our findings,^33^ we spoke with three representatives of charities supporting survival sex workers (SSW) in different regions of England as an exemplar vulnerable group with significant experience of historical and ongoing trauma. They told us how women experience stigma and are afraid of disclosure and confidentiality, particularly if their children have been removed and placed into social care. The charity representatives described how women engaged in SSW rarely sought medical care or achieved registration at a general practice surgery because of lifestyle circumstances and stigmatising experiences.

We asked what trauma-informed care looked like for their service and asked them to reflect on our findings. They recommended a systems-level approach to the delivery of trauma-informed services across the health service. Barriers to access were described as starting at the front door of the general practice surgery with the reaction of the receptionist. A lack of confidentiality in the reception area, closed consulting room doors, short consultation times, and the predominance of digital access methods for appointments were also cited. Beyond these, they suggested responsive, transparent pathways into support services for vulnerable women or those living in extreme circumstances would illustrate a trauma-informed approach to services. Individual practitioners were credited with adopting a trustworthy, trauma-informed approach but charity representatives, in consultation with the women they support, felt that the healthcare system could counteract individual good practice.

## Data

The authors do not have ethical permission to share their dataset beyond the study team.
